# The Therapeutic Uses of Nitro-Glycerine and Nitrate of Amyl*Read before the Medical Association of the State of Alabama, April 14, 1891.

**Published:** 1891-11

**Authors:** S. C. Carson

**Affiliations:** Bessemer, Ala.


### THE THERAPEUTIC USES OF NITRO-GLYCERINE AND NITRATE OF AMYL.*

BY S. C. CARSON, M. D., BESSEMER, ALA.

Life is made up of duties. He who has the most exalted conception of duty, and most nearly approximates that ideal, undoubtedly most accurately and most nobly fills out the full measure of true manhood. My mind never enters this channel of thought without reverting to that unique instance of self-sacrifice so beautifully depicted in "Lucille," where a member of the House of Lords sits patiently day after day—through no love of fame or fortune or honor.

"Then I ask
What inspires and consoles such a self-imposed task
As the life of this man—but the sense of its duty ?
And I swear that the eyes of the haughtiest beauty
Have never inspired in my soul that intense,
Reverential and loving and absolute sense
Of heartfelt admiration I feel for this man
As I see him beside me. - - - - -
His work is the duty to which he was born,
And he accepts it without ostentation or scorn."

These duties become pleasures when, by a process of self-abnegation, we are absorbed as it were, in their performance. While I am conscious that I am more competent to interest you in other fields than that of therapeutics, the mandate of our esteemed President has made it obligatory upon me to write upon

"THE THERAPEUTIC USES OF NITRO-GLYCERINE AND NITRATE OF AMYL,"

a duty that first appeared rather onerous, but gradually lost its irksomeness as each amount of study and research opened up larger and broader areas of thought and richer stores of knowledge. It is true that the text books give but

*Read before the Medical Association of the State of Alabama, April 14, 1891.

little information. The recognized authorities, with the exception of a few—such as Bartholow, Ringer and Brunton—fail to satisfy the student. The journals of the day teem with little items telling of wonderful and magical results in numerous and far from allied diseases. Therefore it is peculiarly gratifying to note the ready aid extended by my friends in the profession—to whom I give most hearty thanks—most especially Dr. John N. Upshur, Professor of Materia Medica in the Medical College of Virginia, who, at my request, prepared the very learned paper on this subject found in the February number of the Virginia Medical Monthly.

I hope to handle my subject at least without *friction*. History has, during past ages, harrowed our souls with the vivid picture of the frailly suspended sword over the head of Democles, in order to impress us with that unheeded fiction—"Uneasy lies the head that wears a crown;" but this pales into insignificance compared to the dread of the present period lest a few grains of nitro-glycerine, surreptitiously deposited beneath his pillow, might hurl the Czar of all the Russias to the four points of the compass. And to those who are addicted to promiscuously prescribing this powerful remedy I will interject a caution by simply remarking that its capacity for harm when administered is only commensurate with its potent external manifestations.

It produces nausea, rapid, weak, dicrotic pulse, gastric pain, sometimes unconsciousness, lowering of temperature, complete revolution of the muscular system of animal life, dilatation of the retinal vessels. The feeble suffer more than the robust. Change in the pulse begins within six minutes and lasts an hour. Motility is first impaired, then sensibility. It lessens sensibility to all forms of irritation and diminishes the reflex functions. It impairs the muscular contractility. Death occurs from failure of respiration; the cerebral functions are only affected when the carbonic acid poisoning ensues. The hæmoglobulin of the blood is damaged, impairing the oxygen-carrying capacity of the red-blood corpuscles, thus accounting for the fall in temperature. The color of the blood is a modified venous hue."—Upshur.

As a very important avenue for obtaining information on my subject I accepted the polite invitation of the manager of the "Sterling Dynamite Company" at Bessemer to visit the factory—the largest in the South—where thousands of pounds of nitro-glycerine, dynamite, black powder, Bessemer powder, etc., are made and sold. I was permitted to see a "run"—that is the mixing of the acids and the glycerine, the nitro-glycerine as it fell to the bottom, again as it was drawn off and washed with soda, as it was worked up with the wood pulp as it was made into "sticks" and loaded into boxes for shipment; in fact, all the minutæ were entertainingly detailed. The acid used is in the proportion of three parts of nitric to four of muriatic. To about 1500 pounds of acid 214 pounds of glycerine are added and the product is about 460 pounds of nitro-glycerine.

As this is a little foreign to the true subject I will only take time to mention one item bearing upon the danger attending the manufacture of these high explosives. The party who alone has charge of the making of the nitro-glycerine is one of three survivors out of fourteen who commenced the process in Pennsylvania fifteen years ago—all others having died violent deaths. I was informed that all the employees suffered more or less in the beginning with headache, but gradually became tolerant of the poison—that the fumes from an explosion were more deleterious than those from the factory.

Some three or four days after this subject was assigned me, as I was sitting in my office—an upper room—several explosions of dynamite occurred in a sewer which was being excavated just below me. I hurried to the door just in time to inhale the fumes as they were wafted upwards. In a very brief time, probably three or four minutes, I remarked to a companion that my head was throbbing from the effects of the smoke. Here was an involuntary experiment upon myself—a point apropos to my subject. Usually I accept the ultimatum of scientists about like I swallow an oyster—that is, whole; but in this instance my own reason began to search out the cause of the head symptoms. The first question then is, how do the nitrates act? Although differing widely in appearance, in stability and in the mode of administration, the effects, which are the result of "nitrous acid being set free in the blood,"are so similar as to readily proclaim their relationship. Their physiological action is to produce "an acceleration of the heart's action, sudden flushing of the face, dilatation of the arterioles in consequence of a paresis of the muscular layer of these vessels, sense of fullness of the brain, with tension and vertigo."—Upshur.

Bartholow says: "All the curative results obtained from nitro-glycerine must be referred to its action upon the vascular apparatus." What action? Do they constrict as Ergot, or do they relax and dilate? Plainly, if there is "a paresis of the muscular layer" there is a relaxation; and clinical experience bears out this idea. This I believe is the true mission of these nitrates, and however wide the range of their seeming applicability their real merit is in diminishing the pressure of their arterioles, whether in temporary contraction or in the more permanent condition produced by sclerosis. It might be of interest here—although not germane to the subject—to inquire into the limit or bounds of contraction and dilatation. It is evident if there is such a thing as forcible dilatation the process *could* go to such an extreme from tonic contraction as to interfere seriously with the heart's action.

According to a well-authenticated law, "there is an inverse ratio between the general blood-pressure and the rate of the pulse." Given a heart overwhelmed by pressure, taxed to its utmost capacity, slowed down to 40 or 50 per minute, then in an instant, by active dilatation, allow the channels to become free, the arterial tone and systemic resistance to be destroyed, a rapid, violent and excited condition of perhaps 150 ensues. Since, then, we cannot deny this power of spasm and expansion to vessels; since we are "so fearfully and wonderfully made,"—a machine so perfect—there must be a regulator to prevent the pendulum from running wild. This centre is by the best authorities located in the medulla. Probably these nitrites have their influence through this special centre. This theory is verified by practice. For instance, in what two affections will you more readily notice the satisfactory results of these remedies than in migraine (of a certain type), and angina pectoris? Quite different, are they not? And yet, see the rational of the same remedy in each. The "slowing of the pulse during an attack of migraine is due probably to cerebral hyperaemia from relaxation of the vessels, or to the secondary anaemia and irritation of the medulla oblongata. This irritation of the medulla is also able to explain the other symptoms of vaso-motor disturbance during an attack of migraine—for instance, the small and contracted radial artery, and the extreme coldness of the feet and hands. Following this stage of irritation of the medulla with contraction of the vessels comes one of exhaustion with relaxation." (Pepper.) Under the head of angina pectoris, Pepper again says: "Various causes have been suggested to account for the seizures, prominent among which is a wide-spread contraction of the arterioles, bringing a sudden strain upon the left ventricle of the heart. This theory is especially noteworthy because of the success which has attended the exhibition of the nitrite of amyl, which brings on a rapid vascular relaxation." Again, I quote from Upshur: "But in angina pectoris—true and pseudo—the achievements of nitrite of amyl are most marked, a few whiffs often bringing prompt relief in those emergent cases where great emergency of symptoms demands the most expeditious action. No agent finds its way into the system with such rapidity except prussic acid. But, though slower somewhat in action, nitro-glycerine even here is more permanently beneficial."

Nitrite of amyl should be administered by inhalation, two, three or five drops on a handkerchief gently wafted under the nostrils of the patient. Nitro-glycerine is given internally, either in the form of liquid nitro-glycerine (1 grain in 100 minims of rectified spirits); dose from one-half to ten minims; pil. nitro-glycerine (1-100 to 1-50 grain in coca butter); dose, one, two or more.

From an interesting little article in the January number of the *American Journal of Medical Sciences* I quote the following on the subject of cardio-vascular vertigo: "It is a symptom of commencing arterio sclerosis—a symptom of temporary disturbance of the circulation of the brain. There exists a sensation of pressure, dyspnoae, on exertion, palpitation, praecordial anxiety, atony of the radial and temporal vessels. The most characteristic symptom is always the accentuation of the aortic second sound. The treatment consists principally in diminishing the tension of the vessel walls ; and for this purpose iod. sodium and nitro-glycerine are indicated—the first in doses of fifteen grains daily; the second in doses of four drops of 1 per cent. solution twice daily, as very valuable for diminishing blood-pressure."

Some five years ago, while practicing in the " black belt," I was taxed with a number of intermittents, accompanied by convulsions. On several occasions I realized the happiest results from a few drops of nitrite of amyl. In this connection Dr. Upshur says there is a fall of temperature, in some cases, of several degrees, following its administration. Often of late I have seen the same remedy highly extolled in chronic hiccough, but I have no personal knowledge in regard to it.

In both acute and chronic Bright's, nitro-glycerine serves a good purpose by relieving vascular tension. Just here I might mention that nitrite of amyl increases the flow of urine similar to alcohol.

It is curious to note the exact similarity between the effects of nitro-glycerine internally and the inhalation of the fumes of dynamite. On this point a most attractive article by Dr. Thos. Barlington can be found in the December No. of the New York *Medical Record.* From 1885 to 1887, while surgeon to the New Croton Aqueduct, 1,300 cases of asphyxia, or partial asphyxia, and poisoning from the products of the explosion of dynamite, came under his care. According to his idea the products of the combustion are water, carbonic acid gas, and nitrogen dioxide. He says: "The similarity of symptoms from inhalation of the products of the explosion of dynamite and of those produced by nitro-glycerine itself is so well marked as to be noticed by the miners themselves. No other conclusion can be reached than the fact that there is mixed with the gases produced, unexploded particles of nitro-glycerine in a volatile state, and these particles, inhaled by the miners, produced the effects described. As regards treatment, of course such measures as are generally used in cases of asphyxia are of service. But in addition to these, the use of cold to the head, and of atropine, ergotine, or other vaso-motor stimulants, administered subcutaneously, are of necessity indicated, and exceedingly efficacious. There is little dou t that the effects of nitro-glycerine are produced from its decomposition and the formation of nitrite in the body. Acting on this principle, and from its stimulant properties, I have uniformly treated my cases with inhalation of ammonia, and also given the carbonate and aromatic spirits of ammonia internally, and up to the present time have never lost a case."

I have been forced to ask myself lately, upon what principle or in what manner, do these agents control pain? I believe all are agreed that they do relieve the fulminant pains of angina; some assert the same as to neuralgia of the fifth pair, of muscular spasm of the stomach, of renal and hepatic colic, of hour-glass contraction of the uterus. If pain is simply "a cry of a nerve for food," and nerve-food is the pabulum of the blood, then probably the question is solved on the ground that more blood, consequently more food, is allowed to reach the part affected. I think it more probable, though, that they paralyze the nerves and destroy the reflexes. On the other hand, how often, during the first quarter of this century, have all pains which "flesh is heir to" been dissipated—like the dew before the sun—by a heroic resort to venesection? Cannot a relationship be established between the two methods? At any rate, I leave this interesting theme to others.

In my early medical career, before my attendance upon societies had sharpened the edge of my unsophisticated perceptions, I imagined that to mention a medical topic in a convention of doctors was to at once rivet the attention of all; that, because it was my food and drink, the words of wisdom and erudition fell upon the ears of all with the same attractive melody as the entrancing stories of the Ancient Montezumas once held my youthful fancies, when the marble palaces, the gilded halls, the resplendent jewels, the graceful senoritas dancing to the music of the mandolin and the low lullaby of fountains gushing forth from beneath the roses, made a picture that yet dwells in my memory. But I am older now. Brevity, besides being the soul of wit, is a much applauded virtue. I seek your grateful plaudits; therefore I close.

